# Cognitive and Neural Aspects of Information Processing in Major Depressive Disorder: An Integrative Perspective

**DOI:** 10.3389/fpsyg.2012.00489

**Published:** 2012-11-12

**Authors:** Lara C. Foland-Ross, Ian H. Gotlib

**Affiliations:** ^1^Department of Psychology, Stanford UniversityStanford, CA, USA

**Keywords:** depression, MDD, neuroimaging, amygdala, interpretation bias, attention bias, memory bias, cognitive control

## Abstract

Researchers using experimental paradigms to examine cognitive processes have demonstrated that Major Depressive Disorder (MDD) is associated not with a general deficit in cognitive functioning, but instead with more specific anomalies in the processing of negatively valenced material. Indeed, cognitive theories of depression posit that negative biases in the processing of information play a critical role in influencing the onset, maintenance, and recurrence of depressive episodes. In this paper we review findings from behavioral studies documenting that MDD is associated with specific difficulties in attentional disengagement from negatively valenced material, with tendencies to interpret information in a negative manner, with deficits in cognitive control in the processing of negative material, and with enhanced memory for negative material. To gain a better understanding of the neurobiological basis of these abnormalities, we also examine findings from functional neuroimaging studies of depression and show that dysfunction in neural systems that subserve emotion processing, inhibition, and attention may underlie and contribute to the deficits in cognition that have been documented in depressed individuals. Finally, we briefly review evidence from studies of children who are at high familial risk for depression that indicates that abnormalities in cognition and neural function are observable before the onset of MDD and, consequently, may represent a risk factor for the development of this disorder. By integrating research from cognitive and neural investigations of depression, we can gain a more comprehensive understanding not only of how cognitive and biological factors interact to affect the onset, maintenance, and course of MDD, but also of how such research can aid in the development of targeted strategies for the prevention and treatment of this debilitating disorder.

## Introduction

Major Depressive Disorder (MDD) is a debilitating psychiatric condition that is characterized by a range of emotional, cognitive, and behavioral symptoms, including core features of persistent depressed mood and decreased interest or pleasure in usually enjoyable activities. MDD is also highly prevalent; almost 20% of the American population will experience at least one clinically significant episode of depression in their lifetime (Kessler and Wang, 2009). Further, more than 75% of these individuals will go on to experience a subsequent episode of depression, often within the first two years of recovery (Boland and Keller, 2009). Given these alarming statistics, it is important for researchers to elucidate the cognitive and neurobiological factors that are involved in the onset, maintenance, and recurrence of this disorder.

Beck’s (1976) cognitive model of depression has advanced our understanding of depression and has served as the foundation for multiple lines of research. This model posits that early adverse events, in combination with other (e.g., genetic, personality) factors, can lead to the development of depressive self-referential schemas that influence information processing through negative biases in attention, memory, and cognition. These biases then confer vulnerability for depression by leading individuals to interpret their experiences in systematically negative ways.

Since the formulation of this model, a wide body of research has amassed examining the cognitive mechanisms of depression and refining cognitive theories of this disorder (Ingram, 1984; Teasdale, 1988; Mathews and MacLeod, 2005). In this paper we review important findings from this work, focusing in particular on research demonstrating that depression is characterized by negative biases in attention, interpretation, memory, and cognitive control. We also present and discuss findings from studies using functional magnetic resonance imaging (fMRI) to elucidate the neural underpinnings of these biases, and review how cognitive and neural dysfunction in depression may influence emotion regulation and inform the development of new treatments. Finally, we briefly discuss the implications of findings of extant research for the prevention of depression.

## Attentional Biases

### Early stages of processing

The question of whether depression is associated with biases during the initial orienting to emotion-relevant information has been examined in studies that use subliminal or very rapid presentations of affective material. For example, in the dot-probe task, two stimuli (one neutral, the other positively or negatively valenced) are presented simultaneously for a brief duration (e.g., 14 ms). A dot then appears in the previous location of one of the two stimuli and participants must indicate the location of the dot as quickly as possible. Biased allocation of subliminal attention to negative emotional material in this task is indicated by faster reaction times to the dot when it appears in the location previously occupied by the negative stimulus. Using this task, Mathews et al. (1996) found no difference in reaction times between clinically depressed and non-depressed participants to anxiety-relevant words. Similarly, Mogg et al. (1995) found evidence for a negative attentional bias in participants diagnosed with an anxiety disorder, but not in participants diagnosed with depression.

These findings, which suggest that depression is not characterized by automatic biases in attention toward negative material, are consistent with results of studies using the subliminal version of the emotional Stroop. In this task, participants are presented with a series of masked emotional and neutral words, and the time participants take to name the non-semantic attributes of the words (most typically the ink color in which a word is presented) is used as a measure of attentional interference caused by the emotional content of the masked words; an increase in reaction time indicates an increase in attention to the emotional meaning of the word. Researchers have largely failed to find a difference between clinically depressed and non-depressed participants’ latency to name the colors of subliminally presented negative words (Mogg et al., 1993; Bradley et al., 1995a; Lim and Kim, 2005). Similarly, Yovel and Mineka (2005) found no associations between biases for subliminally presented depression-relevant words and levels of self-reported depressive symptoms in a sample of undiagnosed undergraduate students.

Finally, a subliminal lexical decision task has been used to examine biases in the rapid orienting to negative material in depression. In this task, participants are presented with a subliminal prime emotional or neutral word, followed by a supraliminal string of letters that participants must judge as being a word or not. Increased attention toward negative material is indicated by shorter response latencies to supraliminal words that are negative in valence and that are identical, or semantically related, to the meaning of the subliminal prime. Using this task, Bradley et al. (1994) found that undiagnosed graduate students with higher levels of negative affect exhibited stronger priming effects for depression-relevant words, and further, that priming effects were more strongly associated with symptoms of depression than with symptoms of anxiety. However, Matthews and Southall (1991) and Dannlowski et al. (2006) failed to observe an effect of subliminal negative primes on reaction times to target words in a sample of clinically depressed individuals.

Although the available behavioral research suggests that major depression is not characterized by biases in the rapid orienting of attention toward negative material, investigators have begun to use neuroimaging methodologies to further explore whether there is neural evidence for biases to subliminally presented stimuli in the absence of observable group differences in behavior. Investigators in this area have focused primarily on patterns of activation in the amygdala, a region that is crucial to the perception of salient affective information and that can process emotional information on a subconscious level by virtue of direct connections from the retina via the superior colliculus and pulvinar (Pessoa, 2005). Results from these investigations are mixed. In one early study, Sheline et al. (2001) observed greater amygdala activation in clinically depressed, relative to non-disordered, participants during the passive viewing of emotional backward-masked faces; importantly, this pattern of hyper-reactivity was not found to be valence-specific. More recently, Dannlowski et al. (2008) found no difference in amygdala reactivity between clinically depressed and non-disordered participants during the viewing of masked sad and angry faces. In contrast, both Suslow et al. (2010) and Victor et al. (2010) found heightened amygdala activation in clinically depressed relative to non-depressed participants during the viewing of masked sad faces. It is important to note, however, that 20% of the depressed participants in Suslow’s study and 50% of Victor’s depressed sample met diagnostic criteria for comorbid anxiety. Therefore, it is not clear whether increased amygdala reactivity in depressed participants in these latter investigations was influenced by this comorbidity.

Considered collectively, there is only modest evidence to support an association between major depression and a subliminal attentional bias toward negative material. While two studies have reported that a neural processing bias may occur in the absence of observable differences in behavior, the influence of anxiety comorbidity clearly requires further investigation. Certainly, distinguishing effects of anxiety from depression on subliminal attention will help to clarify whether these biases are central to the onset and maintenance of depressed mood individuals vulnerable to MDD. Given the high prevalence of comorbid depression and anxiety (Sartorius et al., 1996), however, as well as data showing that the combined disorders are characterized by a more severe course of illness than is either disorder alone (Keller et al., 1983, 1984; Brown et al., 1996), it will be important for researchers to continue to assess patterns of cognitive and neural dysfunction that are associated with comorbid depression and anxiety.

Future research examining automatic processing biases in depression may also benefit from the use of techniques, such as electroencephalography (EEG), that yield high temporal precision. Event-related potentials, for instance, have been used successfully to document differences in the spatial and temporal patterning of neural activity occurring in response to emotional stimuli presented overtly and outside conscious awareness (Williams et al., 2007b). Neuroimaging approaches may also further elucidate the basis for other supraliminal biases in cognition. In a pair of studies, for example, Dannlowski et al. (2007a,b) found that both clinically depressed and non-depressed individuals who exhibited particularly high levels of amygdala activity in response to subliminally presented negative facial expressions, rated neutral material as more negative in a separate series of cognitive assessments conducted outside the scanner than did individuals with low levels of amygdala reactivity. Thus, rapid and automatic patterns of neural responding in the amygdala may contribute to other supraliminal biases in cognition.

### Later stages of processing

In contrast to investigations of attention biases to subliminal stimuli in depression, findings from studies examining biases to negative stimuli that are presented at longer (supraliminal) durations are more consistent in suggesting the presence of a mood-congruent bias in attention. When the presentation of emotional facial expressions is extended in the dot-probe task from 14 to 1000 ms for example, clinically depressed participants, in comparison with non-disordered controls, exhibit longer reaction times to sad than to happy, angry, or fearful faces (Gotlib et al., 2004; Fritzsche et al., 2010). This bias has also been found in formerly depressed participants (Joormann and Gotlib, 2007), as well as in non-disordered girls at familial risk for depression (Joormann et al., 2007b; Kujawa et al., 2011), suggesting that attentional biases are involved in risk for the onset and recurrence of MDD (but see also Mogg et al., 1995; Koster et al., 2006). Based on the results of these and similar studies, researchers have posited that depression is not characterized by a rapid orienting to negative stimuli, but instead, involves difficulties in disengaging from this material once it captures an initial attention (e.g., Gotlib and Joormann, 2010). In line with this formulation, clinically depressed individuals are not found to generally differ from non-disordered controls in their likelihood of attending to negative stimuli, but once their attention is captured by such stimuli, they spend significantly more time looking at this material than do controls (Mathews and Antes, 1992; Eizenman et al., 2003; Caseras et al., 2007; Kellough et al., 2008).

Investigators have documented heightened amygdala activation in clinically depressed individuals during the passive viewing of negative emotional stimuli (Siegle et al., 2002, 2007b; Anand et al., 2005; Dichter et al., 2009; Peluso et al., 2009; but also see Davidson et al., 2003; Irwin et al., 2004), suggesting that attentional biases in the supraliminal processing negative material may be associated, at least in part, with functional abnormalities in this brain region. Moreover, it appears that increased amygdala activation in MDD is sustained beyond the presentation of the negative material itself. For example, Siegle et al. (2002) found that during a valence identification task, whereas non-disordered controls exhibited amygdala responses to all stimuli that decayed within 10 s, clinically depressed individuals were characterized by sustained amygdala responses to negative words that persisted through a subsequent non-emotional distracting task (25 s later) that was designed to induce activation in brain areas hypothesized to suppress activation in the amygdala (e.g., dorsolateral prefrontal cortex; dlPFC). Moreover, sustained amygdala activation to unpleasant words in this study was correlated in depressed participants with self-reported rumination, a hallmark feature of depression that has been posited to be closely linked to sustained processing of negative information. Results that are conceptually similar to findings of sustained amygdala activation in MDD have been obtained in studies examining EEG and pupillary dilation responses to affective material (Deldin et al., 2001; Siegle et al., 2001, 2003; Deveney and Deldin, 2004). Considered collectively, therefore, the available research suggests that depressed persons’ affective experience of negative stimuli persists well beyond the direct exposure to the material and that this processing interferes with engagement in subsequent activities.

Biases in attention to negative stimuli in MDD have also been found in studies using the supraliminal version of the emotional Stroop. Similar to the subliminal version of this task, participants are asked to name the colors in which a series of emotional and neutral words are presented; delays in response times are interpreted as indicating interference caused by increased attention to, and processing of, the emotional meaning of the words. Investigators using the supraliminal emotional Stroop task have found that clinically depressed participants take longer than do non-disordered controls to name the color of negative, but not of positive words (Gotlib and McCann, 1984; Williams and Nulty, 1986; Gotlib and Cane, 1987; Gilboa and Gotlib, 1997). One neuroimaging study has examined patterns of activation occurring during the performance of this task and found that a bias for negative words in clinically depressed individuals was associated with increased activation in the rostral anterior cingulate cortex (rACC) and precuneus (Mitterschiffthaler et al., 2008). This pattern of the results, in the rACC in particular, appear relevant to the study of attentional biases in depression; a large body of research supports a role of this region in the top-down control of attention to emotional material (Bush et al., 2000; Elliott et al., 2000; Vuilleumier et al., 2001). Increased rACC activation in depressed individuals during the processing negative words, therefore, could indicate that these participants require additional activation in this area to be able to override the interference caused by the emotional meaning of these stimuli. The positive correlation that was observed in this study in depressed individuals between activation in the rACC and reaction time latencies to color naming of negative words supports this interpretation. It is also possible, however, that increased activation in the rACC of depressed participants is related more generally to a role of this region in the processing of salient emotional information; the rACC is activated by a variety of affective paradigms, including tasks that involve the generation of sad mood and attention to affective (versus non-affective) characteristics of a pictorial stimuli (Bush et al., 2000; Phan et al., 2004). Thus, it is difficult to determine whether depression-associated anomalies in behavior and brain function during the emotional Stroop are due to negative biases in attention, to negative biases in inhibitory function, or both. The question of whether depression is associated with valence-specific biases in inhibition and cognitive control is an important area of research that we discuss in greater detail below.

Other investigations of possible biases in supraliminal attention to negative material in depression have turned to the use of an emotional version of the Go/No-Go task. In this task, participants are presented with a series of words and are asked to initiate a button press to words that match a target valence and withhold a button press to words that do not. Faster reaction times are interpreted as indicating better detection and, therefore, increased attention to the emotional meaning of the word. Murphy et al. (1999) found that, compared with non-disordered controls, clinically depressed participants were faster during “Go” trials when responding to sad versus happy words. This MDD-related bias toward negative material on the emotional Go/No-Go task has since been replicated (Erickson et al., 2005; Kyte et al., 2005; Kaplan et al., 2006), and been associated in one neuroimaging study with abnormally elevated activation in the rACC (Elliott et al., 2002). Thus, consistent with the neuroimaging literature on the emotional Stroop task, biases in attention toward negative words in the emotional Go/No-Go task appear to be directly related to dysfunction in this region.

One final paradigm that investigators have used to examine the influence of emotion on attention in depression is the emotional oddball task. In a typical oddball experiment, participants are presented with infrequent task-relevant (target) events, to which they initiate a button press, embedded within a continuous stream of frequent standard events. Other task-irrelevant (distractor) events that are equally infrequent are also presented, and the valence of these distractor events is manipulated to assess whether increased attention toward these events influences reaction times to subsequent task-relevant targets. Wang et al. (2008) recently found that, compared with non-disordered control participants, clinically depressed individuals exhibited significant slowing of responses to task-relevant targets when they were preceded by sad, but not by neutral, distractors. Thus, depressed participants appeared more easily distracted by task-irrelevant negative (but not neutral) stimuli than were non-disordered controls, and this distraction interferes with ongoing task performance.

Interestingly, in examining the neural aspects of performance on the emotional oddball task, Wang et al. (2008) found that whereas non-disordered participants’ responses to targets that followed negative (relative to neutral) distractors were associated with activation in the rostral ACC and insula, depressed participants did not show these activations. This difference in activation is important not only in again implicating the rostral ACC in attentional processing biases in depression, but in suggesting more specifically that dysfunction in this region (and in the insula) is associated with, or contributes to, difficulties disengaging from the processing of irrelevant mood-congruent material. What is more, depressed participants in this study demonstrated abnormal elevations in activation of executive (dlPFC) and inhibitory (ventrolateral PFC; vlPFC) regions as they responded to target events that followed sad (but not neutral) distractors. Wang et al. interpreted this pattern of function as compensatory, making up for insufficient levels of activity in the rACC and insula (Harvey et al., 2005; Wagner et al., 2006), although it is also possible that depressed individuals require greater recruitment of the lateral PFC when trying to minimize interference from negative emotional stimuli. Such a formulation is consistent with results of another recent functional neuroimaging study conducted by Kerestes et al. (2012), who found that, compared with their non-depressed counterparts, formerly depressed participants exhibited increased activity in the dlPFC and vlPFC during the presentation of negative, but not of positive, emotional distractors in a working memory task.

Considered collectively, the results of these studies suggest that depression is not characterized a rapid automatic biasing toward negative information, but instead, by difficulties disengaging attention from negative material once it has captured initial attention. Increased processing of negative material by depressed individuals may result from dysfunction in the amygdala. In addition, findings from neuroimaging studies using the Go/No-Go, Stroop, and emotional oddball tasks support the involvement of dysfunctional executive and inhibitory centers in difficulties in attentional control in depression. As we noted above, however, because accurate performance on some of aforementioned tasks depends on intact inhibitory function, it is difficult to determine whether negative biases in attention or in inhibitory function drive neural and behavioral abnormalities. The question of whether depression is associated with valence-specific biases in inhibition and cognitive control is an important area of research that we discuss in greater detail below.

## Interpretation Biases

Investigators have posited that biases in the interpretation of emotionally ambiguous material play a central role in the onset and maintenance of depressed mood. In one early study, Butler and Mathews (1983) examined whether clinically depressed individuals interpret ambiguous information more negatively than do non-disordered individuals. These researchers presented participants with ambiguous social scenarios and then asked them to select, from a set of provided options, which interpretation was the most likely to come to mind. Clinically depressed participants chose the more negative option more frequently than did controls, suggesting the presence of a negative interpretation bias. This finding has since been replicated (Nunn et al., 1997; Voncken et al., 2007). Also consistent with these reports, Mogg et al. (2006), found, using a homophone task in which participants listened to ambiguous words (e.g., die/dye, weak/week) and then wrote the word they heard, that clinically depressed participants tended to write more negative words than did their never-depressed counterparts.

Although informative to our understanding of cognitive functioning in depression, findings of these studies are limited by depression-associated biases in self-report. That is, rather than processing ambiguous material in a more negative manner, depressed persons may have a greater tendency than do their non-depressed peers to report a more negative meaning of scenarios when queried directly about their interpretations. To address this issue, investigators have turned to the use of tasks that involve indirect measures of bias, such as reaction time. Results from these studies, which have generally assessed dysphoric individuals (i.e., individuals experiencing subclinical levels of depression), are mixed. Lawson and MacLeod (1999) presented participants with a series of ambiguous sentences, each of which was followed by a target word that participants read aloud and that was related to either the negative or the neutral interpretation of the sentence. Reaction times to target words were used as an index of interpretation bias: shorter response latencies to negative words than to neutral words were interpreted as being primed by a negative interpretation of the sentence. Results of this study showed no difference between dysphoric and non-dysphoric individuals in reaction times to negative words. This finding was later replicated in a sample of dysphoric participants who received a negative mood induction (Bisson and Sears, 2007), suggesting that response biases were responsible for the negative biases in interpretation reported in previous studies. In another study of dysphoric individuals, however, Lawson et al. (2002) found that dysphoric individuals who exhibited greater severity of depressive symptoms, compared to individuals who were experiencing lower levels of depressive symptoms, showed increased magnitude of the eye blink reflex, a physiological marker of negative emotional processing, during the presentation of ambiguous stimuli. Moreover, using a modified word sentence association paradigm (Beard and Amir, 2009), Hindash and Amir (2012) found that dysphoric individuals were significantly faster to endorse associations between an ambiguous sentence and a negative descriptor word than were non-dysphoric individuals, again supporting an association between depressive symptoms and negative interpretation biases.

Although potentially susceptible to response biases, evidence for a negative interpretation bias in depression also comes from studies using emotion recognition tasks; clinically depressed individuals have been found to be both slower (Leppänen et al., 2004) and less accurate (Leppänen et al., 2004; Gollan et al., 2008) than have non-disordered individuals in recognizing neutral emotions. Moreover, clinically depressed participants have been found to more often misinterpret neutral faces as sad, a bias that has been found to persist following symptom remission (Leppänen et al., 2004). Other investigators have observed that clinically depressed individuals exhibit difficulties in the identification of subtle positive emotion (but no difference in the identification of low-intensity sadness; Suslow et al., 2001; Surguladze et al., 2004; Joormann and Gotlib, 2006). Again, such difficulties have been documented in remitted depressed individuals (Lemoult et al., 2009), and have also been observed in individuals at high risk for depression (Joormann et al., 2010), suggesting that biases in the interpretation of facial emotion represent a risk factor for the onset of a depressive episode.

In summary, studies examining interpretation biases in depression have yielded inconsistent results. This variability in findings may be attributable to biases in self-report, or to the clinical heterogeneity of the participant samples (e.g., sampling from dysphoric versus clinically depressed participants). Certainly, additional studies that examine individuals who are experiencing moderate to severe depression, and that use indirect measures such as reaction time to circumvent potential confounds caused by biases in self-report, are needed to clarify the role of negative interpretation biases in MDD. Moreover, functional neuroimaging studies would help to elucidate the neural correlates of these biases.

## Memory Biases

### Enhanced memory for negative material

One of the most robust and consistent findings regarding cognitive biases in depression involves the preferential recall of negative over positive material (Watkins et al., 1992; Bradley et al., 1995b; Ridout et al., 2003). In a meta-analysis of studies assessing recall performance in clinical depression, Matt et al. (1992) found that individuals with MDD remembered 10% more negative than positive words on explicit recall tasks. Other investigators have found that clinically depressed individuals do not exhibit a bias toward the encoding and recall of positive material that has been reported in non-depressed persons (Gilboa-Schechtman et al., 2002; Ellwart et al., 2003; Harmer et al., 2009b; Gotlib et al., 2011). It is important to point out, however, that these findings have been obtained on tasks of explicit memory; biases in memory for positive and negative stimuli in MDD are found less reliably in studies that assess the unintentional encoding and/or retrieval of valenced material (Matt et al., 1992). This pattern has led researchers to posit that memory biases in depression are due in large part to increased elaboration occurring at later stages of attentional processing (Watkins, 2002). Consistent with this formulation, Koster et al. (2004) found, in a sample of participants experiencing subclinical depression, that attentional biases toward negative words predicted the number of negative words that were subsequently recalled in conditions that allowed for elaborative processing, whereas no such bias was found for words that were presented in conditions that prevented elaborative processing.

Researchers conducting both lesion and functional neuroimaging studies of healthy individuals have found that the amygdala is centrally involved in the encoding and retrieval of emotional material (Cahill et al., 1995; Adolphs et al., 1997). This structure exerts a bottom-up influence on other brain regions, including the hippocampus, which subserve episodic memory formation and retrieval (Steinvorth et al., 2005). Given these findings, neuroimaging studies that have attempted to elucidate the neural underpinnings of negative biases for negative material in explicit memory tasks have focused largely on activations in this region. In the first such study, Ramel et al. (2007) found bilateral amygdala response during the encoding of emotional material predicted increased recall of negative (but not of positive) words in formerly depressed participants in whom a sad mood was induced. This pattern was not observed in non-disordered participants or in remitted depressed participants who did not receive a sad mood induction. In the second study, Hamilton and Gotlib (2008) found that increased memory sensitivity for negative material in clinically depressed individuals was associated with greater activity in the right amygdala during successful encoding of this material. Again, this pattern of activation was not present in healthy participants during the encoding of negative stimuli, or in depressed participants during the encoding of positive stimuli. Interestingly, Hamilton and Gotlib also found that increased activation of the amygdala in depressed individuals was accompanied by increased functional connectivity between the amygdala and the hippocampus, caudate, and putamen, suggesting that during the encoding of negative material, depressed individuals over-recruit a neural network that is involved more generally in enhancing memory for affective stimuli.

### Overgeneral recall of autobiographical memories

In addition to biases in explicit memory, individuals experiencing clinical depression have also been found to demonstrate overgeneral recall of autobiographical memories (see Williams et al., 2007a, for a review). Williams et al. (2007a) argue that reporting overgeneral memories in response to negative cues on the autobiographical memory test (AMT; Williams and Broadbent, 1986) may represent attempts by individuals to protect against the negative affective features that were encoded within the episodic memory system. By recalling overly general descriptions of events that is, one may minimize the experience of negative affect that is attached to distressing memories by blocking access to the details of such memories. Notably, previous studies have found that overgeneral memory in depression correlates with rumination, cognitive deficits, and with longer durations of depressive episodes (Watkins and Teasdale, 2001, 2004; Raes et al., 2005). Further, overgeneral positive memories have been found to predict both poorer recovery from depression (Brittlebank et al., 1993) and a longer delay in recovery from affective disorders (Dalgleish et al., 2001).

Investigators are just beginning to understand the neural correlates of overgeneral autobiographical memory in major depression. In one recent study, Zhu et al. (2012) observed significant associations between patterns of connectivity within the default mode network (DMN) and clinically depressed participants’ tendency to recall overgeneral (versus specific) autobiographical memories: depressed participants who recalled a greater number of overgeneral memories on the AMT exhibited significantly decreased connectivity in the posterior regions of the DMN [posterior cingulate cortex (PCC), precuneus, and angular gyrus]. Interestingly, posterior DMN regions, and in particular the PCC and precuneus, have been found to play a role in the successful retrieval of autobiographical and self-relevant information in non-disordered individuals (Cavanna, 2007; Spreng et al., 2009; Spreng and Grady, 2010). Thus, aberrant activity within, and connectivity between, these regions may contribute to the patterns of overgeneral recall that are observed in depression.

In a second study, Whalley et al. (2012) compared activation patterns between clinically depressed and non-disordered individuals as they viewed cue words referencing a past autobiographical event. Whalley et al. found that although both groups exhibited similar patterns of activation in regions associated with autobiographical memory retrieval (e.g., PCC), depressed individuals showed reduced activity in the vlPFC, an area that has been posited to be a central component to autobiographical networks (Gilboa, 2004; Svoboda et al., 2006). Thus, reduced function in this region could be associated, in addition posterior portions of the DMN, with poorer retrieval of personal memories.

## Deficits in the Cognitive Control of Processing Negative Material

Overriding prepotent responses and inhibiting the processing of irrelevant material that captures attention are core abilities that allow individuals to respond flexibly and to adjust their behavior and emotional responses to changing situations. Deficits in cognitive control and inhibition may be associated with the elaborative and memory biases that we described above that characterize depressed individuals. In this section, we focus on studies that have examined valence-specific abnormalities in cognition involving inhibition and working memory in MDD.

### Inhibition

The negative priming task is one of several experimental tasks that have been used effectively to assess difficulties in cognitive control and inhibition. In this task, participants are asked to respond to a target in the presence of a distractor. For example, on one trial, participants may be asked to name a word written in red ink (target) while ignoring a simultaneously presented word written in blue ink (distractor). Negative priming occurs when inhibition to the blue word remains activated, delaying the response to a target on a subsequent trial if that target is identical to or related to the previously ignored distractor. Thus, delays in responding can be used to assess inhibitory function. In the context of depression, an emotional version of this task, the negative affective priming (NAP) task, has been used to assess depressed individuals’ ability to inhibit the processing of irrelevant negative material. In the NAP task, a negatively valenced distractor on one trial becomes the target on the subsequent trial, allowing investigators to test the hypothesis that if depressed individuals are unable to inhibit the processing of the distractor, they will be faster than non-disordered controls to name the subsequent target. Findings from these studies show that both clinically (Goeleven et al., 2006; Joormann and Gotlib, 2010) and subclinically (Joormann, 2004) depressed individuals respond more quickly than do their non-disordered peers when a negative target is presented following the presentation of a negative distractor. No group differences have been found, however, in reaction times for positive material, suggesting that depressed individuals exhibit difficulties in inhibiting the processing of irrelevant negative, but not of irrelevant positive, material. In addition, difficulties inhibiting negative distractors have been found to be significantly associated with the severity of rumination, even after controlling for level of depressive symptoms (Joormann, 2006; Joormann and Gotlib, 2010).

One functional neuroimaging study of clinically depressed participants has used the NAP task to examine the neural basis of these inhibitory difficulties (Eugène et al., 2010). Results of this investigation showed that problems inhibiting the processing of irrelevant negative material were associated with heightened activity in the rACC of depressed participants. Non-disordered individuals, in contrast, demonstrated heightened activity in this region during the inhibition of positive information. While these findings implicate aberrant functioning of the rACC in difficulties of depressed persons inhibiting the processing of irrelevant negative material, it is important to note that, as was the case in the emotional Stroop task, increased activation in the rACC to irrelevant negative material in depression could reflect either increased salience of this material for the depressed participants (leading to increased conflict when they are required to ignore negative material), and/or difficulties experienced by these participants when they attempt to override automatic responding to these salient stimuli. Indeed, as we discussed above, the latter possibility is consistent with previous work that has implicated the engagement of rACC in the monitoring of emotional conflict (Etkin et al., 2006). Future studies that attempt to tease apart the effects of attention from inhibition with respect to performance on these tasks are needed.

### Working memory

Efficient functioning of working memory systems depends on inhibition, not only to limit the access of information into working memory, but also to remove this information from working memory stores once it is no longer relevant (Hasher and Zacks, 1998). Understanding the biases of working memory systems in depression is important because dysfunctions in expelling negative material from working memory as it becomes irrelevant could lead to difficulties attending to and processing new information and, as a result, to rumination, thereby increasing the likelihood of the onset or maintenance of a depressive episode.

One task that has been used reliably to investigate the role of working memory processes in depression is the emotional Sternberg working memory task (Joormann and Gotlib, 2008). This paradigm requires participants to first memorize two simultaneously presented lists of words. A cue then appears that instructs participants to forget one word list and maintain the other. Finally, a word recognition, or “probe,” epoch is presented when participants must indicate whether the probe word came from the list they were previously cued to remember, from the list they were cued to forget (i.e., the no longer relevant list), or did not come from either list. The difference in reaction times to an intrusion probe (i.e., a probe from the irrelevant list) and reaction times to a new probe (i.e., a completely new word) reflects the strength of the residual activation of the contents of working memory that were declared to be no longer relevant and, therefore, assesses a person’s ability to remove irrelevant information from working memory (Oberauer, 2001). By manipulating the valence of the relevant and irrelevant word lists, as is the case in the emotional Sternberg working memory task, investigators can assess participants’ ability to remove negative and positive material from working memory.

Using this task, Joormann and Gotlib (2008) found that clinically depressed individuals exhibit longer decision latencies than do their non-disordered counterparts to negative (but not to positive or neutral) probe words from the no longer relevant word lists. This finding was subsequently replicated (Berman et al., 2011), and is consistent with the formulation that depression is associated with difficulties in disengaging from negative information that was once, but is no longer relevant. This valence-specific deficit in working memory may help to explain why depressed individuals respond to negative life events with recurring, uncontrollable, and unintentional negative thoughts. Consistent with this possibility, Joormann and Gotlib (2008) found that deficits in the removal of no longer relevant negative information from working memory were associated with the severity of rumination, a finding that remained significant even when controlling for depressive symptoms.

Our group recently adapted the emotional Sternberg task for use in a functional neuroimaging investigation to examine whether difficulties removing negative information from working memory in depression were associated with anomalous neural function (Foland-Ross et al., in press). Results from our investigation indicated that during the presentation of the instruction cue, when participants are required to forget one word list and maintain the other, clinically depressed individuals showed greater activation than did non-disordered control participants in the dorsal anterior cingulate cortex (dACC), superior parietal lobule (SPL), and insular cortices when expelling negative information. In contrast, control participants exhibited stronger activation in these regions when attempting to expel positive material. Interestingly, the dACC, SPL, and insular regions are important components of the task positive network (TPN), a collection of structures that has been postulated to subserve active cognitive processing (e.g., executive control and working memory; Fox et al., 2005). Moreover, activation in these structures has been documented to increase linearly with cognitive effort (Paus et al., 1998; Wager et al., 2005; Allen et al., 2007). Depressed individuals, therefore, may exert greater cognitive and neural effort than do their non-depressed counterparts in removing no longer relevant negative material from working memory.

While these studies demonstrate that depressed individuals are less able than are non-depressed persons to expel negative information from working memory, thereby exacerbating the effects of negative content on cognition, other investigators have examined whether depressed individuals are also impaired at selecting relevant positive material for processing in working memory. Difficulties in this area could limit depressed persons’ ability to use positive material to ameliorate the adverse effects of processing negative information. Levens and Gotlib (2009) examined this possibility using an emotional version of the recency-probes task. In this task, participants are presented with a set of three target words, followed by a brief fixation, and then a probe display, in which a single word is presented. Participants are asked to indicate, using a button press, whether the probe word was previously presented as one of three words in the target set. Individuals’ ability to successfully select material for processing in working memory is assessed using reaction times to probes during interference trials, in which the probe word is not a member of the target set for that trial, but was a member of previous target sets. Longer response times during these trials reflect impairments in the selection of that material for initial processing in working memory. Levens and Gotlib found that, compared to non-disordered controls, clinically depressed individuals exhibited longer response times during interference trials for positive (but not for negative or neutral) stimuli, indicating that depressed persons are impaired in selecting task-relevant positive stimuli for processing in working memory.

In sum, the results of research examining cognitive inhibitory functioning in depression suggest that depressed individuals experience problems both inhibiting the access of irrelevant negative material into working memory and disengaging from this material in working memory once it is no longer relevant. In addition, depressed individuals do not show the normative bias toward the selection and maintenance of positive material demonstrated by non-depressed individuals. Difficulties in the control of working memory contents, in turn, appear related to dysfunctioning of the rACC as well as components of the TPN. Below, we review research that has attempted to link cognitive difficulties and biases to the dysregulation of emotion that has been found to characterize individuals diagnosed with MDD.

## Effects of Cognitive Biases on Emotion Regulation in Depression

Cognitive deficits and biases in the processing of emotional information are likely to impair depressed individuals’ ability to adaptively regulate their emotions. Below, we discuss how biases in attention and memory, and difficulties in cognitive control, might affect emotion regulation processes in depression, and contribute to persistent negative mood.

### Attention

Arguably one of the easiest ways to regulate emotion is to try to look away from or ignore an emotion-eliciting stimulus or situation. Individual differences in attention for emotional information, therefore, represent one important factor that may influence emotional states. As we have described earlier in this paper, depression does not appear to be characterized by a rapid automatic biasing toward negative information. Rather, once this material captures initial attention, individuals experiencing clinical depression have difficulty disengaging from this material. This tendency of being “stuck” in attending to negative aspects of the situation may prevent depressed people from using effective emotion regulation strategies such as distraction and attentional avoidance when confronted with stressful events. Moreover, biases in attention may interfere with a person’s ability to successfully reframe emotion-eliciting events and lead to the sustained processing of emotion-eliciting stimuli and prolonged negative affect.

Several studies of non-disordered samples have investigated whether difficulties disengaging from negative information can increase emotion reactivity and impair emotion regulation efforts. Compton et al. (2000) reported that decreased ability to disengage attention from negative stimuli was related to increased reactivity to an upsetting film. Similarly, Ellenbogen et al. (2006) found that problems disengaging attention from dysphoric images was associated with increased negative affective in response to a subsequent stressor. MacLeod and Hagan (1992) extended these findings by demonstrating that women with the strongest attentional biases toward negative material reported the greatest increases in distress following a diagnosis of cancer. Given this research, it will be important in future studies for investigators to examine, using causal experimental designs, whether attentional biases have deleterious effects on emotional reactivity in individuals with depression.

It will also be important to examine the neural relations between attention and emotional experience. Van Reekum et al. (2007) found that, when cognitively reappraising negative scenes, non-disordered participants tended to shift their gaze away from the extremities of an image, and that individual differences in gaze fixation accounted for a significant portion of variance in neural activation of the amygdala. These findings suggest not only that effortful forms of emotion regulation rely, at least in part, on attention, but further, that the ability to disengage attention from emotion-inducing aspects of a situation can directly influence activity in brain regions that work to mediate emotional responses. Whether there are similar relations among emotion, gaze, and neural function in depression is unknown.

### Interpretation of emotionally ambiguous information

Individuals often impose meaning on ambiguous situations without intervening awareness of possible alternative interpretations. An increase in the tendency to interpret ambiguous situations as negative could influence vulnerability to experience negative mood. Evidence in support of this postulation comes from studies of non-clinical samples that have examined the relation between interpretation biases and prospective measures of mood symptoms and emotional reactivity. Rude et al. (2002) found that greater negative interpretation bias in non-disordered participants was significantly associated with an increase in depressive symptoms at a follow-up assessment. In a subsequent study, Holmes et al. (2009) observed that training positive biases in non-disordered individuals helped to alleviate the effects of an induced negative mood state. Training a negative interpretation bias, in turn, was found by Wilson et al. (2006) to predict heightened emotional reactivity to a subsequent stressful video clip. Thus, the tendency to interpret ambiguous scenarios in a negative or positive manner likely influences the generation and experience of an emotional response.

### Memory

Biases in memory can also influence emotion regulation in significant ways. It is well documented, for example, that recalling positive autobiographical memories can repair negative mood in non-disordered individuals (Josephson et al., 1996; Rusting and Dehart, 2000; Joormann and Siemer, 2004). Increased memory sensitivity for negative material in depression, therefore, could contribute to the difficulties for depressed individuals to access mood-incongruent (i.e., positive) material, causing interference with the selection and use of positive memories to regulate emotion. Consistent with this formulation, Josephson et al. (1996) found that, whereas non-clinical samples of undergraduate students who scored low on measures of depression severity tended to retrieve positive memories following sad mood induction, individuals reporting more severe negative mood symptoms were more likely to retrieve mood-congruent memories. Students in this study, in turn, who engaged in mood-incongruent recall following a negative mood induction showed the greatest improvement in subsequent mood.

Other studies suggest that clinically depressed individuals are as able as their non-disordered peers to recall positive memories, but are less effective in using these memories to repair negative mood states. For example, Joormann et al. (2007a) had clinically depressed, remitted depressed, and never-depressed participants report levels of affect following the induction of a sad mood, and again after they wrote a list of positive autobiographical events. Joormann et al. found that, although the groups did not differ in the number, intensity, or specificity of positive autobiographical events that they recalled, whereas never-depressed participants’ mood improved following the recall of positive memories, the sad mood of remitted and currently depressed participants remained unchanged or had worsened.

Reduced specificity of autobiographical memory may also influence emotion regulation in depression. As we note above, overgeneral memory represents one avoidance strategy that may be useful in minimizing distress in the short term (Raes et al., 2003). In the longer term, however, overgeneral memory places individuals at increased risk for developing depression (Gibbs and Rude, 2004). Although the precise reasons for this process are unclear, theorists have speculated that avoiding the recall of specific aspects of past events hinders the development of effective strategies for solving interpersonal problems (Evans et al., 1992). Poor problem-solving abilities, in turn, could lead to more negative social encounters in the future (Hermans et al., 2005), and contribute to increased negative mood.

### Cognitive control

Biases in cognitive control may also contribute to emotion-regulatory difficulties in depression. One common type of emotion regulation strategy that is both highly dependent on cognitive control mechanisms and that is effective in altering the trajectory of an unfolding emotional response is cognitive reappraisal. This approach to emotion regulation involves cognitively reframing the meaning of a stimulus or event in a manner that makes the event less distressing. Individuals who more frequently use cognitive reappraisal have fewer depressive symptoms than do individuals who use other emotion regulation strategies, such as emotion suppression and rumination (Gross and John, 2003; Campbell-Sills et al., 2006; Garnefski and Kraaij, 2006, 2007). Neuroimaging studies demonstrate that engagement in cognitive reappraisal strategies is associated with increases in activation of the dACC and ventro- and dorsolateral PFC, and with decreases in activation of areas that mediate emotional responding, such as the amygdala (Ochsner et al., 2002, 2004). This inverse coupling between frontal and subcortical limbic areas is consistent with the formulation that reappraisal triggers top-down cognitive control of emotion-generating systems. Moreover, the finding that reappraisal activates the same regions that are activated in working memory and inhibition – areas that are dysfunctional in depression (Elliott et al., 2002; Beauregard et al., 2006; Johnstone et al., 2007; Mitterschiffthaler et al., 2008; Erk et al., 2010; Eugène et al., 2010; Kerestes et al., 2012) – supports the position that emotion regulation depends on cognitive functioning in these areas, and that dysfunction in these regions, paired with difficulties in controlling negative, mood-congruent contents of working memory, may directly impair depressed individuals’ ability to flexibly reappraise or reinterpret life events or situations (Siemer, 2005, 2007).

Investigators have just begun to examine neural correlates of effortful emotion regulation in depression. Beauregard et al. (2006) found that clinically depressed individuals experienced more difficulty in regulating emotional responses to a sad film clip than did never-depressed participants, and that this difficulty was related to increased activity in the dACC. Similarly, Johnstone et al. (2007) found that, during the cognitive reappraisal of negative images, non-disordered individuals exhibited an inverse correlation between activation in the amygdala and vmPFC. In contrast, clinically depressed participants demonstrated a positive correlation between these regions, as well as heightened activation in a separate, more dorsal, portion of the PFC, suggesting that effortful attempts to reappraise negative situations in depression are counterproductive. And finally, in a cognitive reappraisal study reported by Erk et al. (2010), clinically depressed individuals were found to be able to down-regulate amygdala responses; however, this effect was short-lived and was associated with decreased activation of the dlPFC. Taken together, although replication studies are certainly needed, a picture of neural dysfunction is beginning to emerge in depression indicating that emotion regulation in this disorder is associated with abnormalities in prefrontal function.

Importantly, negative biases in inhibitory and working memory function may contribute to the occurrence of rumination. Ruminative responding – or the recurrent, self-reflective, and unintentional focus on depressive symptoms and their possible causes and consequences (Nolen-Hoeksema, 1991) – has been significantly associated with difficulties in the ability of depressed persons to inhibit, expel or disengage from negative cognitions, and memories that were once, but are no longer, relevant (Joormann, 2006; Joormann and Gotlib, 2008, 2010). Rumination, in turn, may exacerbate negative emotional states; in a study of individuals experiencing subclinical depression, Williams and Moulds (2010) found that participants who were induced to ruminate reported more negative mood and rated intrusive memories as more distressing than did dysphoric participants who were distracted. Moreover, in a recent review, Nolen-Hoeksema et al. (2008) reported evidence suggesting that a greater tendency to ruminate, when combined with negative cognitive biases, predicts longer episodes of depression. Clearly, therefore, a vicious cycle can emerge between deficits in cognitive control and negative mood states, and represents an important area for further research.

## Treatment Implications

The cognitive biases and deficits that have been found to characterize depression may represent important targets for intervention. Indeed, given findings demonstrating that biases in attention, memory, and cognitive control are associated with course of illness (e.g., Johnson et al., 2007), ameliorating dysfunction at the cognitive level may help to reduce depressive symptomatology, as well as other features of depression, such as rumination. As we review in this section, a small, but growing body of research has found that cognitive biases can be targeted directly by computer-based training paradigms, and that this training is effective in reducing depressive symptoms.

### Attention

Investigators attempting to manipulate attentional biases in depression have used a modified dot-probe task. In the training version of this paradigm, a pair of stimuli are presented to the participant, but the subsequent probe is presented more frequently in the location of the neutral or positive stimulus than in the location of the negative stimulus, thereby directing participants’ attention away from the negative stimulus and toward the neutral or positive stimulus. This attentional training procedure has been found to reduce subsequent emotional reactivity to induced or to real-life stressors in undiagnosed participants (MacLeod et al., 2002; Dandeneau and Baldwin, 2004; Dandeneau et al., 2007). In two recent experiments of individuals experiencing clinical and subclinical depression, Baert et al. (2010) examined the effects of attentional bias modification on depressive symptomatology. Importantly, given that depression appears to be characterized by biases that occur in later stages of stimulus processing (see Teachman et al., 2012 for a review), Baert et al. had participants perform a dot-probe training task in which stimuli were presented at supraliminal durations (e.g., 1500 ms). Results of these investigations showed that 2 weeks of training away from negative and toward positive stimuli led to improvements in depressive symptomatology in mildly depressed participants; however, participants with more severe symptoms of depression did not show such improvement. The reasons for this latter finding are not clear, and it is important for investigators to examine more explicitly the mechanisms underlying the apparent differential effectiveness of attentional bias training.

In another study of mild to moderately depressed participants, Wells and Beevers (2010) examined the effects of four sessions of attention training away from negative and toward neutral stimuli, again using relatively longer stimulus durations to allow participants to more fully process the content of the stimuli. These investigators found that four sessions of training over a period of 2 weeks resulted in a significant and immediate decrease in depressive symptoms that was not present in participants who received sham training. Moreover, these reductions were found to persist in a 2-week follow-up assessment. Interestingly, path analyses showed that symptom improvement was mediated by attention modification training, suggesting that the manipulation of this bias directly affected symptom levels.

### Interpretation of emotionally ambiguous information

Few studies have examined whether modifying negative interpretation biases can have a significant impact on negative affect in depressed individuals, despite demonstrations that computerized cognitive bias modification (CBM) techniques that target interpretation (CBM-I) can cause a significant increase in the tendency for non-disordered participants to interpret new and emotionally ambiguous information in a positive manner (Holmes et al., 2006, 2009). In this training approach, individuals are repeatedly presented with potentially ambiguous scenarios whose interpretation is constrained in a particular direction (positive or negative). In non-disordered samples, repeated positive CBM-I training sessions have been found to reduce vulnerability to anxiety to a later stressor (MacKintosh et al., 2006). Further, positive CBM-I has been found to be effective in reducing negative interpretative biases in individuals who report high levels of anxiety (Mathews et al., 2007; Murphy et al., 2007; Salemink et al., 2009).

Only one study to date has examined the effects of positive CBM-I training in depression. Blackwell and Holmes (2010) had depressed individuals listen to a series of ambiguous situations in which ambiguity was resolved in a positive direction. Following daily sessions of positive CBM-I training for 1 week, four out of seven of the depressed participants demonstrated significant improvements in depressive symptoms. Moreover, these effects were maintained at follow-up, 2 weeks later. Although modest, such a response rate is comparable to that of studies examining efficacy of antidepressant medication or CBT (Hollon et al., 2002). Thus, more rigorous testing using larger samples with a sham control group is warranted.

### Memory

Given studies showing that overgeneral memory influences the course of depression (Brittlebank et al., 1993; Dalgleish et al., 2001; Watkins and Teasdale, 2001, 2004; Raes et al., 2005), investigators have also begun to examine the effects of interventions designed to increase memory sensitivity. Watkins et al. (2009) found that training participants with subclinical depression to actively engage in generating concrete construals (e.g., focusing on specific details of an event) when thinking about autobiographical memories led to significant reductions in both depressive symptoms and frequency of rumination. In another study of more severely depressed participants, Raes et al. (2009) found that training clinically depressed inpatients to recall more specific memories generated improvements in their memory retrieval, level of rumination, and quality of problem-solving. Thus, overgeneral memory may represent another cognitive aspect of depression that, like attention, is amenable to successful modification.

Researchers have also examined whether biases in explicit memory are amenable to training. In a study investigating whether individuals could be trained to forget negative material using a modified “Think-No-Think” (TNT) paradigm (Anderson and Green, 2001), Joormann et al. (2009) presented clinically depressed and non-disordered participants with word pairs consisting of one neutral cue word and one positive or negative target word. In an initial learning phase, participants were asked to learn cue-target associations. Participants were then instructed not to think about the negative targets when shown their neutral cues, but instead, to either think of a new valenced word (substitute condition) or to refrain from thinking of another word (unaided condition). Joormann et al. found that, when asked to recall the original target words, depressed participants in the substitute, but not in the unaided condition showed successful forgetting of negative words. Moreover, the number of negative targets they forgot was directly related to length of training. Thus, although this study examined memory performance within a single session and did not assess symptom change, these findings are promising in suggesting that future training paradigms could be developed, using modified versions of the TNT task, to help depressed individuals suppress their enhanced retrieval of negative material.

### Cognitive control

Although difficulties in controlling the access and manipulation of negative material into working memory represent clear features of cognitive disturbance in depression, few studies have examined whether modifying this domain of cognition can have beneficial effects on depressive symptomatology. Indeed, in the only study that has examined the effects of cognitive control training on depression, Siegle et al. (2007a) had clinically depressed and non-disordered participants undergo 2 weeks of training using a pair of tasks: a variant of the Paced Auditory Serial Attention Task (Gronwall, 1977), in which individuals continuously add serially presented digits in working memory, and an attention task (Wells, 2000) that requires participants to selectively attend to specific environmental auditory stimuli. Results of this study showed that mood symptoms decreased continuously in clinically depressed individuals throughout the two-weeks of intervention, as did rumination. Moreover, analyses of neural data from a subsample of the depressed participants in this study revealed that cognitive control training was associated with a reduction in amygdala reactivity to negative affective stimuli, as well as an increase in dlPFC activity during the performance of a working memory task. Given these initial findings, as well data from several studies of dementia indicating that cognitive control training has a significant, although unexpected, effect of lowering depressive symptoms (see Sitzer et al., 2006 for a review), the potential impact of cognitive control training on depressive symptoms and diagnosis, and on levels of ruminative thinking, is promising.

### Treatment implications: Summary

The available research suggests that depression-associated biases in attention, memory, interpretation, and cognitive control may be targeted directly using computer-based training paradigms, and that attenuating these biases may effectively reduce severity of depressive symptoms. Such training approaches could serve as useful monotherapies, or alternatively, may be used as adjunctive tools for other pharmacologic and therapeutic interventions. Given evidence that information processing biases may be present prior to the initial onset of depression (Joormann et al., 2007b, 2010), future investigations should examine whether early interventions with cognitive training could prevent the onset of MDD through inhibiting or reversing the development of dysfunctional schemas. At the same time, because some bias modification procedures have been found to be effective only in individuals who are experiencing mild symptoms of depression (e.g., Baert et al., 2010), investigations examining the parameters of effective training programs for specific types or severity of depression would be helpful to prevention efforts.

## Summary and Future Directions

As we have documented in this review, the available research finds that a diagnosis of MDD is accompanied by increased elaboration of negative information, a tendency to interpret ambiguous information as negative, difficulties disengaging from negative material, and deficits in cognitive control when processing this material. Neuroimaging investigations of the neural bases of these difficulties find, in the context of biases in attention, that sustained processing of negative affective material in depression is associated both with prolonged activation of the amygdala and dysfunction in the rACC. Examinations of the neural underpinnings of enhanced memory for negative material in depression, in turn, appear to be associated with the over-recruitment of the amygdala, as well as other subcortical regions that are involved more generally in the encoding of affective material. And finally, problems in controlling and inhibiting the processing of negative material in depression relates to dysfunction in higher-order cognitive control regions, including the ACC, dlPFC, and vlPFC.

Several important goals for future research remain. One involves the more systematic mapping of links between cognitive and affective aspects of depression. It will be critical, for example, for researchers to elucidate the causal nature of the relation between depressed mood and cognition. This issue could be addressed through studies that continue to investigate the impact of cognitive retraining on emotion, or through longitudinal investigations that examine whether cognitive biases observed during a clinically significant episode of depression resolve following successful treatment with medication. Along these lines, it would be helpful for future studies to understand the mechanisms by which cognitive retraining improves mood; it is currently unclear, for example, whether retraining attention biases in depression alters symptom profiles through altering bottom-up processes involved in generation of affect, top-down processes involved in the regulation of affect, or through influencing other aspects of cognitive functioning (e.g., memory).

In addressing possible interactions between cognitive biases, investigators have begun discussions on the “near” and “far” transfer of training effects of CBM (Hertel and Mathews, 2011; MacLeod and Mathews, 2012). While bias modification training typically transfers to new stimulus materials presented in assessment versions of a task (near transfer), changes in emotional functioning or performance on tasks that are less closely related to the training task (far transfer) are particularly informative for our understanding of mechanisms. Findings from a small of collection of recent investigations indicate that far transfer effects occur with training that is focused on discrete aspects of cognition. One instance of this comes from a study by White et al. (2011), who found that attention bias training leads to changes in interpretive biases. Similarly, training of interpretation biases have been found to influence biases in both attention (Amir et al., 2010) and memory (Salemink et al., 2010; Tran et al., 2011). These findings suggest not only that the distinctions made by researchers among biases in attention, interpretation, and memory need to be reconsidered, but further, that at least some aspects of these biases share common mechanisms of action.

Future studies will also benefit from the integration of cognitive biases with other biological aspects of depression. A number of recent studies have found that reductions in serotonin levels, induced by tryptophan depletion, causes cognitive impairments in non-disordered individuals that are similar to those found in depression. This includes reduced autobiographical memory specificity (Alhaj et al., 2012), selective encoding of negative material (Wang et al., 2009), and attention toward negative stimuli (Murphy et al., 2002; Roiser et al., 2008). Moreover, similar to clinically depressed individuals, increased amygdala responses to negative material (Roiser et al., 2008), and altered activation in ventrolateral prefrontal regions during the processing of task-irrelevant negative distractors (Wang et al., 2009) are also found in participants treated using acute tryptophan depleting compounds. Further support for the involvement of serotonin in neural and cognitive abnormalities in depression comes from studies examining associations between these measures and polymorphisms of the serotonin transporter-linked polymorphic region (5-HTTLPR): compared to long-allele homozygotes, individuals who carry one or two 5-HTTLPR short alleles have been found to exhibit difficulties disengaging attention from emotional stimuli (Beevers et al., 2009) and to have greater amygdala reactivity to sad material (Hariri et al., 2005). Given these findings, it follows that cognitive biases in depression may be modulated, at least in part, by serotonergic systems, and that negative affective biases are amenable to treatment with medications that target this system (e.g., SSRIs; Clark et al., 2009; Harmer et al., 2009a). To date, however, investigators have not examined changes in cognitive biases in depressed individuals as a consequence of pharmacological treatment. Certainly, an investigation into the interaction between serotonin and cognition represents an area for promising research in depression. Along these lines, studies examining the relation between cognition and other aspects of neurobiological function implicated in depression and in the regulation of affective states (e.g., dopamine, cortisol) would be helpful in the developing a more complete understanding of the cognitive abnormalities that are associated with MDD.

Finally, a small, but growing body of literature is demonstrating that depression-related biases in cognition are not necessarily correlates or consequences of the experience of depression, but could reflect a pattern of dysfunction that precedes the initial onset of this disorder. Indeed, like depressed adults, young individuals who are not themselves depressed but are at high risk for developing depression by virtue of having a depressed parent have been found to demonstrate negative biases in the interpretation (Dearing and Gotlib, 2009) and identification (Joormann et al., 2010) of neutral and emotional material. Moreover, similar to depressed adults, never-disordered girls at familial risk for depression selectively attend to negative facial expressions on the dot-probe task (Joormann et al., 2007b). It is possible, therefore, that negative biases in cognition play a direct role in placing children of depressed parents at increased risk for developing a depressive disorder. A necessary next step to addressing this question involves using longitudinal designs to examine whether specific cognitive biases can predict which high risk individuals are at greatest risk for developing depression. Similarly, given neuroimaging findings that familial risk for depression is associated with depression-related abnormalities in brain structure (Peterson et al., 2009; Chen et al., 2010; Rao et al., 2010; Huang et al., 2011) and function (Monk et al., 2008; Mannie et al., 2011), it would be helpful if future studies could examine whether abnormalities in neural measures are useful in predicting the onset of depression. Furthermore, and perhaps most importantly, given the initial promise of cognitive retraining procedures in altering cognitive biases in adults with depression, researchers need to develop a more comprehensive understanding of how cognitive retraining might be used in the prevention of major depression.

In reviewing the literature on cognitive biases in depression, we have described several sources of methodological variability that, if addressed, may increase the consistency in the results of future studies. First, despite recent estimates that up to 65% of depressed individuals experience clinically significant symptoms of anxiety (Brown et al., 2001), the effects of this comorbidity on the neural and behavioral measures of depression are not fully understood. Although current evidence suggests that trait levels of anxiety may account for biases in subliminal attention that have been reported in some studies of clinical depression (Gotlib and Joormann, 2010; Teachman et al., 2012), additional research is needed examining the effects of this comorbidity on automatic perception and other aspects of cognition and neural function. Second, cognitive biases in depression may be influenced by the duration of MDD; individuals who are experiencing a first episode of depression have been found to exhibit a different pattern of emotional responding than do individuals who have experienced recurrent episodes of depression (e.g., Nandrino et al., 2004). Related to this issue are findings showing that cognitive and neural processing biases in depression (Suslow et al., 2010) are affected by depression severity (e.g., Dannlowski et al., 2007a; Suslow et al., 2010). Third, there is evidence to suggest that task performance varies as a function of how depressed individuals process experimental stimuli. Some investigators, for example, have found depressed individuals to exhibit difficulties inhibiting the processing of mood-congruent material, but only when this material was processed in a self-referential manner (Segal et al., 1995; Power et al., 2000). Along these lines, there is also evidence showing that stimulus type (e.g., words versus images) influences task performance (Isaac et al., 2012). Fourth, future investigations of depression are likely to benefit from researchers controlling for trauma history. Childhood maltreatment is a significant predictor of both amygdala response (Dannlowski et al., in press), and biases in attention (Fani et al., 2010) in adult participants. Moreover, epidemiological studies show that trauma occurring during childhood is associated with the onset and severity of depressive symptoms during adulthood (see Heim et al., 2008 for a review). Fifth, the effects of gender on depression-related abnormalities in neural and cognitive is still unclear; investigators often combine men and women in their samples or sample exclusively from women. Given that men and women have different clinical manifestations of depression (Piccinelli and Wilkinson, 2000; Nolen-Hoeksema, 2001), studies examining how emotional material is processed in depressed females versus males would be helpful in elucidating the parameters of cognitive and neural biases in major depression. Such research could also help to explain the gender difference in the prevalence of MDD (e.g., Nolen-Hoeksema and Hilt, 2009). Finally, investigators should examine more closely and control for the effects of psychotropic medication on cognition and neural function. Given the findings we presented earlier showing that targeted manipulation of the serotonergic system using tryptophan depletion leads to cognitive and neural changes in non-disordered adults that resemble those found in major depression, the issue of exactly how SSRIs or medications targeting other neurotransmitter systems may affect neurocognitive profiles of depressed adults clearly warrants further research.

In closing, although there are several issues that require further study, the available research indicates that MDD is characterized by significant impairments in the processing of mood-congruent material in the context of attention, interpretation, memory, and cognitive control, and that these cognitive difficulties contribute to difficulties in emotion regulation and neural dysfunction. Investigators have also provided important new leads in the development of alternative treatments for depression in the form of computer-based cognitive bias retraining paradigms, which may be helpful as adjuncts to existing therapies or as prevention strategies in individuals at high risk for developing this disorder. It will be important to continue to integrate findings across different research modalities in order to inform etiological models of depression and advance the development of new approaches to the prevention and treatment of this debilitating disorder.

## Conflict of Interest Statement

The authors declare that the research was conducted in the absence of any commercial or financial relationships that could be construed as a potential conflict of interest.
